# Impact of early response on outcomes in AL amyloidosis following treatment with frontline Bortezomib

**DOI:** 10.1038/s41408-021-00510-7

**Published:** 2021-06-21

**Authors:** Sriram Ravichandran, Oliver C. Cohen, Steven Law, Darren Foard, Marianna Fontana, Ana Martinez-Naharro, Carol Whelan, Julian D. Gillmore, Helen J. Lachmann, Sajitha Sachchithanantham, Shameem Mahmood, Philip N. Hawkins, Ashutosh D. Wechalekar

**Affiliations:** National Amyloidosis Centre, University College London (Royal Free Campus), London, UK

**Keywords:** Myeloma, Prognosis

## Abstract

The outcomes in systemic AL amyloidosis are dependent on the depth of haematologic response. However, there is limited data on the impact of the speed of response on outcomes. Here we report the impact of speed of response in a cohort of AL patients treated with upfront Bortezomib. Patients seen from February 2010 until August 2019 are included in the present analysis. 1194 & 1133 patients comprised the ITT and 1-month landmark cohorts. In the landmark cohort, 137 (11.5%), 270 (22.6%), 252 (21.1%) and 352 (31.1%) patients had a CR, VGPR, PR and NR at 1-month. Patients with ≥ VGPR at 1-month had significantly better survival (median not reached; at the end of 1, 2, 5,10 years, 87%/92%, 83%/87%, 68%/72% and 63%/58% of patients in CR/VGPR, respectively, were alive) compared to those with a PR (median OS 60 months) or NR (median OS 32 months) (*p* < 0.005). At 1-month, patients with CR and iFLC < 20 mg/l had a significantly better survival compared to CR and iFLC > 20 mg/l (*p* = 0.005). Reaching ≥ VGPR at 1-month significantly improved survival in all Mayo disease stages. In conclusion, patients achieving an early deep haematologic response have a significantly superior survival irrespective of cardiac involvement.

## Introduction

Systemic immunoglobulin light chain amyloidosis (AL) is a rare disorder of protein misfolding caused by an underlying monoclonal B-cell or plasma cell dyscrasia. About two-thirds of patients have cardiac involvement at diagnosis, the most important prognostic determinant [1]. The amyloidogenic immunoglobulin-free light chain is the determinant of the disease. In the absence of any proven therapies to remove the deposited fibril protein, treatment remains focused on clonal suppression. With the introduction of novel anti-plasma cell therapy, there has been a marked improvement in the survival of AL patients over the last decade [2]. Bortezomib-based regimes are the current first-line standard of care. While achieving a profound suppression of the amyloidogenic light chain has been shown to correlate with better survival [3–5], the impact of the speed of response on outcomes remains less well studied. In a selected group of AL patients, the Greek Amyloidosis group and we have previously shown that achieving an early response is associated with better outcomes [6, 7]. Conceivably, a slower response could be associated with organ progression before the response is achieved and adversely impact outcomes, whilst a rapid response may avoid this.

In this study, we report the impact of speed of response on outcomes in a large cohort of unselected AL patients treated with upfront Bortezomib-based regimes.

## Patients and methods

### Patients

All patients from the ALchemy study (an ongoing prospective observational study of newly diagnosed AL amyloidosis) seen at the UK National Amyloidosis Centre from February 2010 until August 2019 and treated with upfront Bortezomib are included in the present analysis. The ALchemy study is approved by the relevant institutional review board, and all patients provided written informed consent per the declaration of Helsinki. Patients with a difference between involved and uninvolved free light chain (dFLC) < 20 mg/l at diagnosis were excluded due to a lack of validated response criteria in this patient group [8, 9].

Details of the investigations at diagnosis/follow-up, schedule of follow-up at NAC and treatments is available in the supplementary appendix.

Organ involvement and responses were defined by ICC [10, 11]. The European modification of Mayo 2004 staging was used, with Stage III stratified into IIIa (NT-proBNP < 8500 ng/L) and IIIb (NT-proBNP ≥ 8500 ng/L) [12]. We also collected data on response assessment, survival status, and dates of death/last known follow-up.

We report the responses based on the validated criteria reported by Palladini et al. [3, 13]. As per the standard follow-up protocol, serum-free light chain (FLC) and serum monoclonal protein levels (when present) were monitored at the beginning of each chemotherapy cycle. In this study, we report haematologic responses at 1, 3 and 6 months and outcomes based on these responses. Organ responses are reported based on the criteria published by Palladini et al. [3]. and Gertz et al. [11].

Due to challenges in postal transport of monthly urine samples, we defined responses at 1 and 3 months based on serum immunofixation and serum-FLCs alone. The response was defined according to the validated criteria at 6 months (including urine immunofixation). Very good partial response (VGPR) was defined as a dFLC < 40 mg/l, and partial response (PR) was defined as a 50% decrease in the dFLC over the baseline. Patients with a < 50% decrease in dFLC over baseline were classified as no response (NR). Recent studies have shown that patients achieving a deep suppression of the amyloidogenic light chain have a superior response [4, 5]. Therefore, we also identified patients who achieved a dFLC < 10 mg/l and involved free light chain (iFLC) < 20 mg/l.

Overall survival (OS) was calculated from the date of diagnosis to the time of death, or the last known follow-up. Patients who were alive were censored at the date of their last known follow-up.

### Statistical analysis

We performed all statistical analysis using SPSS version 27 (IBM Inc, USA). The method of Kaplan and Meier was used to generate survival curves. The two-sided log-rank test was used to assess statistical significance between survivals. All reported p values are two-sided with the conventional significance of ≤0.05. The independent *t*-test was used to test for a significant difference in means between the subjects. We used regression models (Cox proportional hazards model and binary logistic regression) to analyse the impact of early haematologic response on survival and organ function. We analysed all data on an intent to treat basis. We also performed a landmark analysis of survivors at 1, 3 and 6 months. Organ responses were assessed in the 12-month landmark cohort.

## Results

**Fig. 1:**
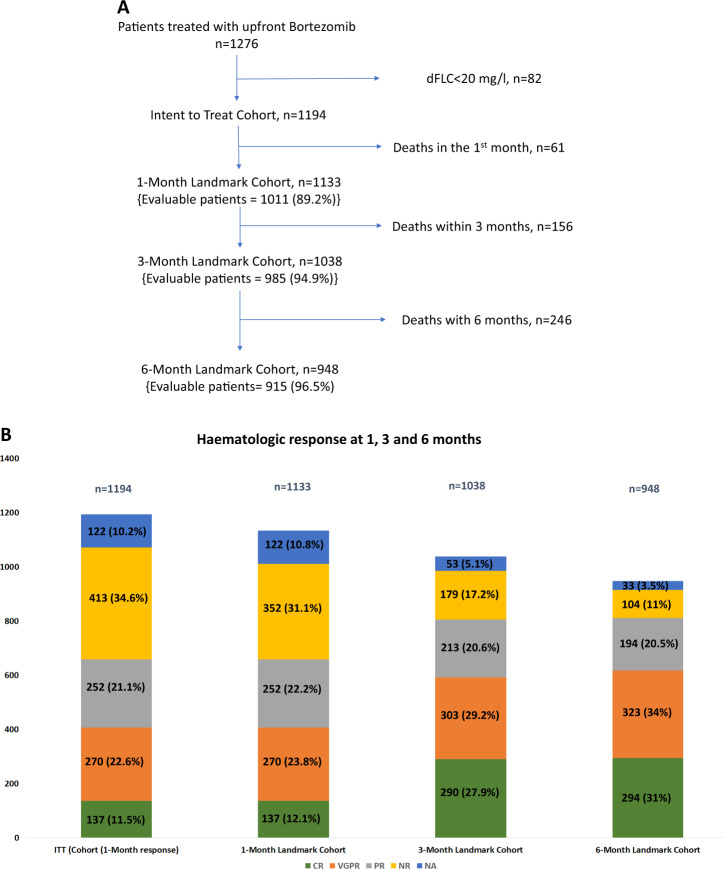
This figure shows the flow of patients in the study and responses acheived at different time points in the study. **A** Consort diagram. 1276 patients were treated with upfront Bortezomib from Feb 2010 to Aug 2019. 82/1276 (6.4%) had dFLC < 20 mg/l and are excluded from the analysis. There are 1194, 1133, 1038, and 948 patients in the ITT, 1-month, 3-month and 6-month landmark cohorts. **B** Shows the haematologic responses 1 month, 3 months and 6 months. In the ITT cohort 137 (11.5%), 270 (22.6%), 252 (21.1%) and 413 (34.6%) patients had a CR, VGPR, PR and NR at 1 month, respectively. There was a progressive improvement in the proportion of patients with a deeper response (≥ VGPR) from 34.1% at one month to 57.1% and 65% at 3 and 6 months, respectively.

A total of 1276 patients received upfront Bortezomib from Feb 2010 until the end of Aug 2019. Eighty-two patients had dFLC < 20 mg/l at diagnosis and are excluded from this analysis. 1194 patients comprise the intention to treat cohort (ITT cohort). Table 1 lists the baseline characteristics of these patients. 61, 156 and 246 patients died within 1, 3 and 6 months of diagnosis. 1133, 1038 and 948 patients comprised the 1-month, 3-month and 6-month landmark cohorts, respectively. 1011 (89.2%), 985 (94.9%) and 915 (96.5%) patients were evaluable in the respective cohorts (Fig. 1A).

**Table 1:** Baseline characteristics.

	*N* = 1194
Baseline characteristics	Median (range) or *n/N* (%)
Age, y	66 (29–88)
Gender	Male: 713 (59.7); Female: 481 (40.3)
Performance status	ECOG 0-2: 1117 (93.6)
	ECOG > 2: 77 (6.4)
Cardiac involvement	791 (66.2)
Mayo stage (European modification of Mayo stage)	I: 183 (15.3)
	II: 409 (34.3)
	IIIa: 418 (35)
	IIIb: 184 (15.4)
NT-proBNP, ng/L	1393 (4–93602)
High-sensitivity cardiac troponin T, ng/L	57 (1–742)
Left ventricular wall thickness, mm	13 (8–24)
Renal involvement	802 (67.3)
Serum creatinine, µmol/L	96 (26–1124)
Proteinuria, g/24 h	2.9 (0–36)
Liver involvement	139 (11.6)
Alkaline Phosphatase, U/L	90 (16–2389)
GI Involvement	48 (4)
Autonomic nervous system involvement	82 (6.9)
Peripheral nervous system involvement	85 (7.1)
Soft tissue involvement	187 (15.7)
Heavy chain isotype	IgG: 386 (32.3)
	IgA: 161 (13.5)
	IgM: 38 (3.2)
	IgD: 9 (0.8)
	LC: 312 (26.1)
	None: 288 (24.1)
Serum monoclonal protein, g/L	8 (IF-45)
Light chain isotype	Kappa: 258 (21.6)
	Lambda: 936 (78.4)
dFLC, mg/L	208.5 (20.1–15898)

*dFLC* the difference between involved and uninvolved light chains, *ECOG* Eastern co-operative oncology group, *NT-proBNP* N-terminal pro-brain natriuretic peptide

The median age was 66 years (range 29–88) years. 66.2%, 67.3% and 11.6% of patients had cardiac, renal, and liver involvement, respectively. The median dFLC, NT- proBNP and proteinuria were 208.5 mg/l (range 20.1-15898 mg/L), 1393 ng/l (range 4–93602 ng/L) and 2.9 gm/ 24 h (range 0–36 g/24 h), respectively. Patients received a median of six cycles of chemotherapy (range 1–12 cycles). Eighty-seven patients underwent an autologous stem cell transplant (ASCT).

### Haematologic response

The haematologic responses at 1, 3, and 6 months are shown in Fig. 1B. In the ITT cohort 137 (11.5%), 270 (22.6%), 252 (21.1%), and 413 (34.6%) patients had a CR, VGPR, PR and NR at 1 month, respectively. There was a progressive improvement in the proportion of patients with a deeper response (≥VGPR) from 34.1% at one month to 57.1% and 65% at 3 and 6 months, respectively.

### Impact of speed and depth of response on overall survival

**Fig. 2:**
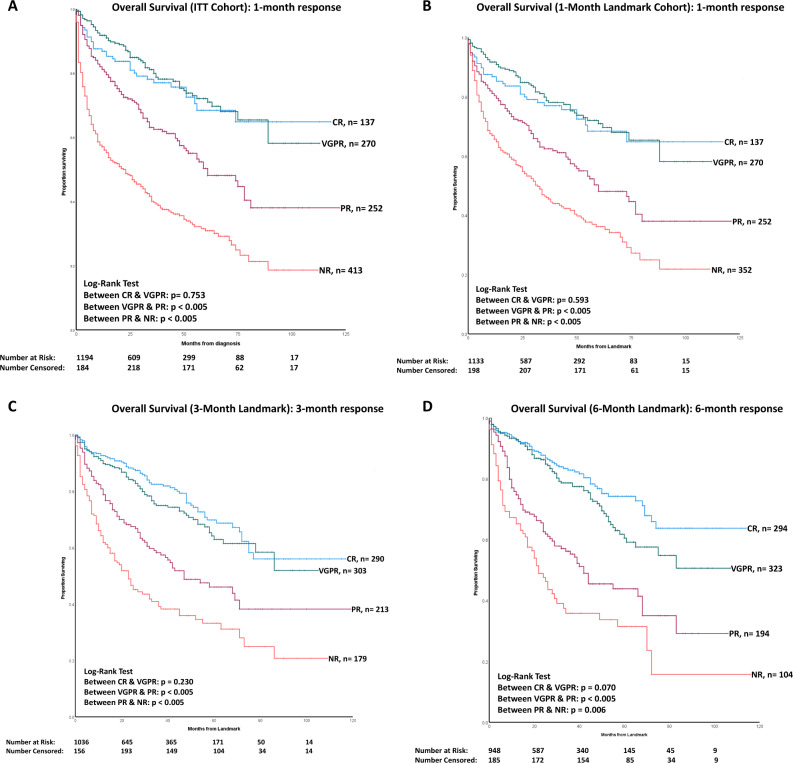
This figure shows overall survival for the intention to treat (ITT), 1 month, 3 months and 6 months landmark cohorts stratified by depth of haematologic response. **A** Kaplan–Meier curve showing the impact of 1-month haematologic response on OS in the ITT cohort. There was no significant difference in survival between CR and VGPR at 1 month—median not reached in both groups (*p* = 0.753). 87%, 83%, 68% and 63% of patients with a CR at 1 month were alive at the end of 1, 2, 5 and 10 years, respectively. 92%, 87%, 71% and 59% of patients with a VGPR at 1 month were alive at the end of 1, 2, 5 and 10 years, respectively. In contrast, the median OS of patients with PR and NR at 1 month was 61 months (95% CI 43.42–78.57 months, *p* < 0.005), and 22 months (95% CI 14.54–29.45 months, *P* < 0.005), respectively. **B** Kaplan–Meier curve showing the impact of 1-month haematologic response on OS in the 1-month landmark cohort. There was no significant difference in survival between CR and VGPR at 1 month—median not reached in both groups (*p* = 0.593). 87%, 83%, 68% and 63% of patients with a CR at 1 month were alive at the end of 1, 2, 5 and 10 years (from landmark point), respectively. 92%, 87%, 72% and 58% of patients with a VGPR at 1 month were alive at the end of 1, 2, 5 and 10 years (from landmark point), respectively. In contrast, the median OS of patients with PR and NR at 1 month was 60 months (95% CI 42.42–77.57 months, *p* < 0.005) and 32 months (95% CI 25.36–38.63 months, *P* < 0.005), respectively. **C** Kaplan–Meier curve showing overall survival based on the haematologic response at 3 months in the 3-month landmark cohort. There was no significant difference in survival between CR and VGPR at 3 months—median not reached in both groups (*p* = 0.230). 93%, 88%, 69% and 55% of patients with a CR at 3 months were alive at the end of 1, 2, 5 and 10 years (from landmark point), respectively. 91%, 84%, 65% and 51% of patients with a VGPR at 3 months were alive at the end of 1, 2, 5 and 9 years (from landmark point), respectively. In contrast, the median OS of patients with PR and NR at 3 months was 47 months (95% CI 27.51–66.48 months, *p* < 0.005) and 23 months (95% CI 15.93–30.06 months, *p* < 0.005), respectively. **D** Kaplan–Meier curve showing overall survival based on the haematologic response at 6 months in the 6-month landmark cohort. There was no significant difference in survival between CR and VGPR at 6 months—median not reached in both groups (*p* = 0.070). 93%, 88%, 74% and 63% of patients with a CR at 6 months were alive at the end of 1, 2, 5 and 10 years (from landmark point), respectively. 93%, 86%, 61% and 51% of patients with a VGPR at 6 months were alive at the end of 1, 2, 5 and 10 years (from landmark point), respectively. In contrast, the median OS of patients with PR and NR at 6 months was 42 months (95% CI 27.91–56.09 months, *p* < 0.005) and 22 months (95% CI 16.39–27.60 months, *p* = 0.006), respectively.

Table 2 shows patients’ overall survival in the ITT, 1-month, 3-month and 6-month landmark cohorts, stratified by their haematologic response. The median OS of the ITT cohort was 56 months (95% CI 47.02-64.97 months). In the ITT cohort, at 1-month, patients with a CR or VGPR had significantly better survival than those with a PR or NR (*p* < 0.005) (Fig. 2A). Similar results were seen at 3 and 6 months (Figs. SA1 and SA2 in supplementary appendix). In the landmark cohorts (1, 3 and 6 months), the median OS of patients achieving a CR or VGPR was significantly better than those with a partial or no response (*p* < 0.005) (Fig. 2B–D). There was no significant difference in survival between patients with CR and VGPR in all cohorts- median OS not reached in both groups in all cohorts, *p* = 0.753 (ITT), *p* = 0.593 (1-month landmark), *p* = 0,230 (3-month landmark) and *p* = 0.070 (6-month landmark), respectively.

**Table 2:** Overall survival stratified by haematologic response- ITT, 1-month, 3-month and 6-month landmark cohorts.

	CR	VGPR	PR	NR	*p* value
	*n*	*n*	*n*	*n*	
	Median OS, months (95% CI, months)	Median OS, months (95% CI, months)	Median OS, months (95% CI, months)	Median OS, months (95% CI, months)	
ITT cohort Evaluable patients = 1194	*n* = 137	*n* = 270	*n* = 252	*n* = 413	<0.005
	Median not reached	Median not reached	Median OS 61 months (95% CI 43.42–78.57 months)	Median OS 22 months (95% CI 14.54–29.45 months)	
1-month landmark cohort Evaluable patients = 1011	*n* = 137	*n* = 270	*n* = 252	*n* = 352	<0.005
	Median not reached	Median not reached	Median OS 60 months (95% CI 42.42–77.57 months)	Median OS 32 months (95% CI 25.36-38.63 months	
3-month landmark cohort Evaluable patients = 985	*n* = 290	*n* = 303	*n* = 213	*n* = 179	<0.005
	Median not reached	Median not reached	Median OS 47 months (95% CI 27.51–66.48 months)	Median OS 23 months (95% CI 15.93–30.06 months	
6-month landmark cohort Evaluable patients = 915	*n* = 294	*n* = 323	*n* = 194	*n* = 104	<0.005
	Median not reached	Median not reached	Median OS 42 months (95% CI 27.91–56.09 months)	Median OS 22 months (95% CI 16.39–27.60 months)	

*CR* complete response, *VGPR* very good partial response, *PR* partial response, *NR* no response

### Early deep response (≥VGPR at 1-month) versus late deep response (≥VGPR at 6 months): impact on overall survival

Since a deep haematologic response at 1 month following treatment impacted outcomes, we analysed the survival of patients achieving an early deep response (at 1 month) and compared them with those who had not achieved this response at 1 month but had reached a deep response later (at 6 months). There were 915 evaluable patients with response data at 1 and 6 months. 501 patients (54.75%) had not achieved a CR or VGPR at 1-month. 224/501 (44.71%) patients improved their response to CR or VGPR at 6 months. Using the Cox proportional hazards model, patients who achieved a deep haematologic response early had significantly better survival than those who achieved a deep haematologic response later- HR 1.454, 95% CI 1.039–2.033 (*p* = 0.029). The median OS was not reached in the early responders versus 74 months in the late responders (*p* = 0.027) (Fig. SA3 in supplementary appendix).

### Impact of deep suppression of the amyloidogenic light chain on outcomes

**Fig. 3:**
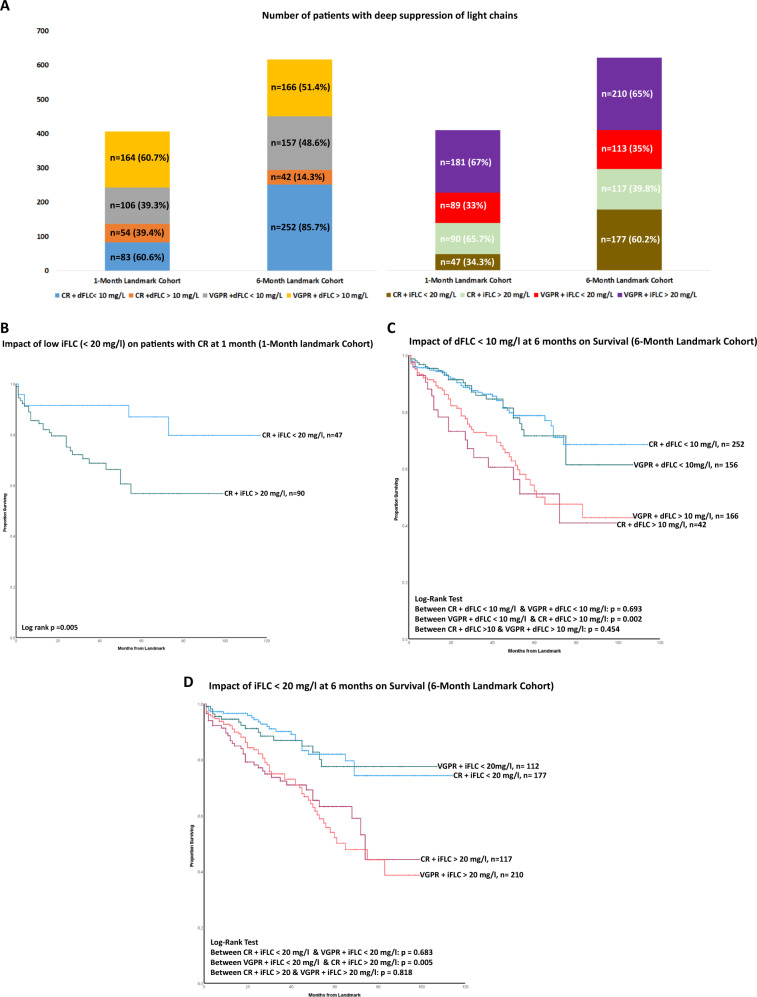
This figure shows patients acheiving a deep light chains suppresion (dFLC <10 mg/L or iFLC <20 mg/L) and outcomes stratified by deep light chain suppression (or not). **A** Shows the distribution of patients with CR/VGPR and deep FLC suppression (dFLC < 10 mg/l or iFLC < 20 mg/l) at 1 and 6 months. At 1 month, 60.6% and 39.3% patients with CR and VGPR had dFLC < 10 mg/l, respectively. The corresponding figures at 6 months were 85.7% and 48.6%, respectively. 34.3% and 33% of patients with CR and VGPR had iFLC < 20 mg/l at 1 month. The corresponding figure at 6 months is 60.2% and 35%, respectively. **B** Kaplan–Meier curve showing the impact of low iFLC (< 20 mg/l) on patients with CR at 1-month. Patients with CR and iFLC < 20 mg/l at 1-month had significantly better survival compared to patients with CR and iFLC > 20 mg/l- median not reached in both groups (*p* = 0.005). **C** Kaplan–Meier curve showing the impact of dFLC < 10 mg/l in patients with CR/VGPR at 6 months. There was no significant difference in survival between patients with CR + dFLC < 10 mg/l and VGPR + dFLC < 10 mg/l—median not reached in both groups (*p* = 0.693). Patients with VGPR + dFLC < 10 mg/l had a significantly better survival when compared to patients with CR and dFLC > 10 mg/l (*p* = 0.002)—median not reached vs. 72 months (95% CI 38.94–105.05 months). There was no significant difference in survival between patients with CR + dFLC > 10 mg/l and VGPR + dFLC > 10 mg/l (*p* = 0.454)—median 72 months (95% CI 38.94–105.05 months) vs. 61 months (95% CI 38.07-83.93 months). **D** Kaplan–Meier curve showing the impact of iFLC < 20 mg/l in patients with CR/VGPR at 6 months. There was no significant difference in survival between patients with CR + iFLC < 20 mg/l and VGPR + iFLC < 20 mg/l—median not reached in both groups (*p* = 0.683). Patients with VGPR + iFLC < 20 mg/l had a significantly better survival when compared to patients with CR and iFLC > 20 mg/l (*p* = 0.005)—median not reached vs. 74 months (95% CI 65.60–82.40). There was no significant difference in survival between patients with CR + iFLC > 20 mg/l and VGPR + iFLC > 20 mg/l (*p* = 0.818)—median 74 months (95% CI 65.60–82.40) vs. 65 months (95% CI 46.81–83.18 months).

We also analysed outcomes in CR/VGPR patients based on the degree of suppression of the amyloidogenic light chain (dFLC < 10 mg/l and iFLC < 20 mg/l). Figure 3A shows the distribution of patients with CR/VGPR and dFLC < 10 mg/l / iFLC < 20 mg/l at 1 and 6 months.

In the 1-month landmark cohort, patients with CR and iFLC < 20 mg/l at 1-month had significantly better survival compared to patients with CR and iFLC > 20 mg/l—median not reached in both groups (*p* = 0.005) (Fig. 3B). There was no significant difference in survival of CR at 1-month based on dFLC < or > 10 mg/L (Fig. SA4 in supplementary appendix). Also, there was no significant difference in survival of VGPR at 1-month based on their dFLC or iFLC level.

In the 6-month landmark cohort, we analysed patients’ survival stratified by the following variables- CR, less than CR but dFLC < 10 mg/l or iFLC < 20 mg/l. The survival of patients with CR (*n* = 294) was compared to those who were not in CR but had dFLC < 10 mg/l (*n* = 155) or iFLC < 20 mg/l (*n* = 111). There was no significant difference in survival between patients with CR and those not in CR but had a dFLC < 10 mg/l—median OS not reached in both groups (*p* = 0.617) (Fig. SA5 in supplementary appendix). Similarly, there was no significant difference in survival between patients with CR and those not in CR but had achieved an iFLC < 20 mg/l—median OS not reached in both groups (*p* = 0.233) (Fig. SA6 in supplementary appendix).

Finally, for a more granular understanding of how deep suppression of the amyloidogenic light chain affects survival, we compared the following groups of patients based on their dFLC - CR + dFLC < 10 mg/l, VGPR + dFLC < 10 mg/l, CR + dFLC > 10 mg/l, VGPR + dFLC > 10 mg/l; and iFLC levels – CR + iFLC < 20 mg/l, VGPR + iFLC < 20 mg/l, CR + iFLC > 20 mg/l and VGPR + iFLC > 20 mg/l (Fig. 3C, D).

Patients with CR or VGPR and dFLC < 10 mg/l had a significantly better survival compared to CR or VGPR and dFLC > 10 mg/l (*p* < 0.005). There was no significant difference in survival between patients with CR + dFLC < 10 mg/l and VGPR + dFLC < 10 mg/l—median not reached in both groups (*p* = 0.693). Patients with VGPR and dFLC < 10 mg/l had a significantly better survival when compared to CR and dFLC > 10 mg/l (*p* = 0.002)—median not reached vs. 72 months (95% CI 38.94–105.05 months) (Fig. 3C).

Similarly, patients with a CR or VGPR and iFLC < 20 mg/l had a significantly better survival compared to CR or VGPR and iFLC > 20 mg/l (*p* < 0.005). There was no significant difference in survival between patients with CR + iFLC < 20 mg/l and VGPR + iFLC < 20 mg/l—median not reached in both groups (*p* = 0.683). Patients with VGPR + iFLC < 20 mg/l had a significantly better survival when compared to CR and iFLC > 20 mg/l (*p* = 0.005)—median not reached vs. 74 months (95% CI 65.60–82.40) (Fig. 3D).

### Impact of early response on overall survival stratified by Mayo disease stage

**Fig. 4:**
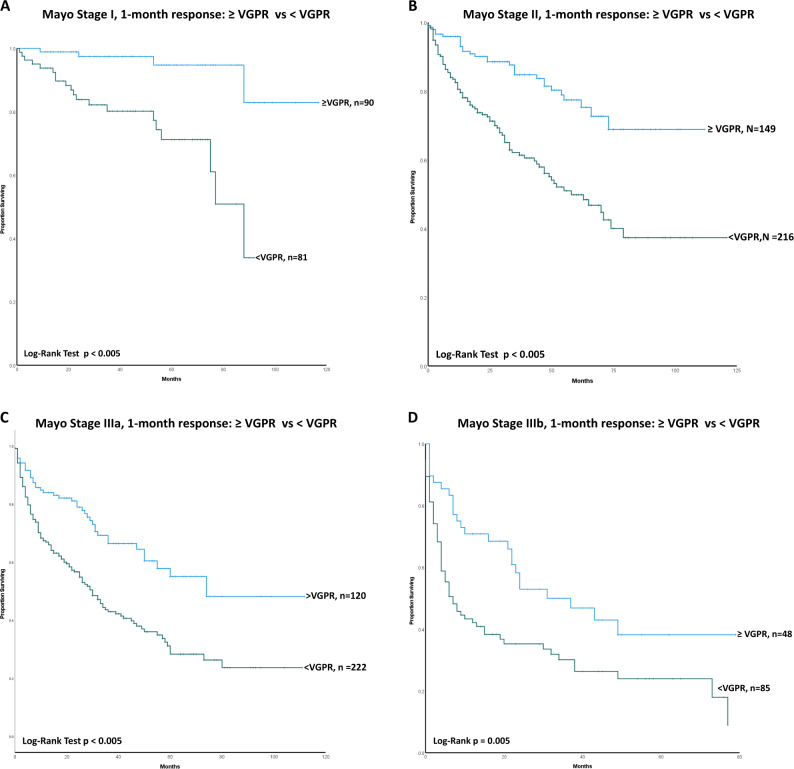
This figure shows the impact of early response on survival stratified by baseline disease (Mayo) stage showing an improvement in outcomes with deeper responses across all stages. **A** Kaplan–Meier curve showing overall survival of Mayo stage 1 patients, stratified by their haematologic response at 1 month (≥ VGPR vs < VGPR). The median OS of patients with ≥ VGPR was not reached when compared to 88 months (95% CI 72.65–103.35 months) in patients with < VGPR (*P* < 0.005). **B** Kaplan–Meier curve showing overall survival of Mayo stage 2 patients, stratified by their haematologic response at 1 month (≥ VGPR vs < VGPR). The median OS of patients with ≥ VGPR was not reached when compared to 58 months (95% CI 41–74.99 months) in patients with < VGPR (*P* < .005). **C** Kaplan–Meier curve showing overall survival of Mayo stage 3 patients, stratified by their haematologic response at 1 month (≥ VGPR vs < VGPR). The median OS of patients with ≥ VGPR was median 74 months when compared to 30 months (95% CI 23.69–36.30 months) in patients with < VGPR (*P* < 0.005). **D** Kaplan–Meier curve showing overall survival of Mayo stage 3B patients, stratified by their haematologic response at 1 month (≥ VGPR vs < VGPR). The median OS of patients with ≥ VGPR was 31 months (95% CI 11.05–50.95 months) when compared to 7 months (95% CI 3.03–10.96 months) in patients with < VGPR (*P* < 0.005).

We analysed outcomes based on 1-month response (1-month landmark cohort) stratified by the Mayo stage. As we found no significant difference between OS for patients with CR and VGPR, they were analysed as a group and compared to patients with less than a VGPR (PR or NR). We found that patients with ≥ VGPR at 1-month had significantly better survival than patients with < VGPR (*p* < 0.005). The improved survival was seen across all Mayo stages. The median OS of patients achieving ≥ VGPR vs < VGPR was: Mayo stage I: median not reached vs was 88 months (95% CI 72.65–103.35 months); Mayo stage II: median not reached vs 58 months (95% CI 41–74.99 months); Mayo stage IIIa median 74 months vs 30 months (95% CI 23.69–36.30 months) and Stage III b: median 31 months (95% CI 11.05–50.95 months) vs 7 months (95% CI 3.03–10.96 months), respectively (Fig. 4A–D). Of patients with Mayo stage I disease reaching ≥ VGPR at 1 month, 99%, 99%, 94% and 82% were alive at the end of 1, 2, 5 and 10 years (from landmark point). Similarly, 96%, 90%, 77% and 68% of Mayo stage II patients with ≥ VGPR at 1 month were alive at the end of 1, 2, 5 and 10 years (from landmark point).

### Impact of early deep response on organ function

Finally, we analysed the impact of early haematologic response on organ function in the 12-month landmark cohort. 841 patients were available for analysis in the 12-month landmark cohort. 498 (59.2%) patients had cardiac involvement, and 586 (69.7%) had renal involvement. As there were only 90 (10.7%) patients with liver involvement, we have not analysed the liver response in the present cohort.

Cardiac response was evaluable in 394/498 (79%) and 356/498 (71.5%) patients at 6 and 12 months, respectively. 103 (26.1%) and 194 (54.5%) patients had a cardiac response at 6 and 12 months, respectively. 188 (37.8%) and 269 (54%) patients had ≥ VGPR and < VGPR at 1-month, respectively. At 6 months, 51/188 (27.1%) patients with ≥ VGPR at 1-month had a cardiac response compared to 44/269 (16.4%) patients with < VGPR. At 12 months, 87/188 (46.3%) patients with ≥ VGPR at 1-month had a cardiac response compared to 94/269 (34.9%) patients with < VGPR.

Renal response was evaluable in 449/586 (76.6%) and 458/586 (78.2%) patients at 6 and 12 months, respectively. 99 (22%) and 115 (25.2%) of patients had renal response at 6 and 12 months, respectively. 266/586 (45.4%) and 287/586 (49%) patients had ≥ VGPR and < VGPR, respectively. At 6 months, 55/266 (20.7%) patients with ≥ VGPR at 1-month had a renal response compared to 41/287 (14.3%) patients with < VGPR. At 12 months, 80/266 (30.1%) patients with ≥ VGPR at 1-month had a renal response compared to 31/287 (10.8%) patients with < VGPR.

Patients who had ≥ VGPR at 1 month had significantly higher odds of having a cardiac (OR 1.839, 95% CI 1.147–2.948, *p* = 0.011) or renal (OR 1.577, 95% CI 0.997–2.492, *p* = 0.05) response at 6 months. Similarly, patients who had ≥ VGPR at 1 month had significantly higher odds of having a cardiac (OR 1.565, 95% CI 1.005–2.437, *p* = 0.047) or renal (OR 3.560, 95% CI 2.227–5.693, *P* < 0.005) response at 12 months.

We also compared the odds of organ response at 12 months in patients with early deep response and those who achieve a deep response later. There was no significant difference in the odds of cardiac response at 12 months between early and late responders—OR 1.294, 95% CI 0.770–2.174, *p* = 0.330. However, early responders had significantly better odds of achieving a renal response at 12 months than late responders—OR 2.790, 95% CI 1.565–4.973, *p* = 0.001.

## Discussion

This study assesses the impact of early clonal response on survival in a large, unselected cohort of patients with AL amyloidosis treated with frontline bortezomib. We show that patients who achieve a deep clonal response (≥ VGPR) within 1 month have a significantly better outcome that appears to be driven by deep suppression of the amyloidogenic light chain. Patients who achieve an early deep response have a superior survival and better organ responses than those who achieve a deep response later. The key finding is that benefit of rapid response is seen across all disease stages.

In systemic AL amyloidosis, a rapid reduction in the amyloidogenic light chains is desirable to stop the amyloidogenic pathway early. Studies in myeloma have shown that patients who respond early have a poorer outcome [14–16], possibly due to suppression of a highly proliferative plasma cell population in the marrow [17], prone to clonal evolution, leading to an early relapse. However, AL amyloidosis, with usually a modest plasmacytosis and different biology of the plasma cells, this may not be the case [3, 18, 19].

There is limited data on the impact of rapid response on outcomes in AL amyloidosis. In two small studies, including a highly selected patient population, we showed that patients achieving an early and deep haematologic response have better overall survival and renal outcome, respectively [6, 7]. A study by the Greek amyloidosis group [20] suggested that deep haematologic response within one month had a median OS of 9.5 years vs 3.9 years and 1.8 years for patients with PR or NR, respectively. The present study extends these results showing that a deep and rapid haematologic response improves outcomes with or without cardiac involvement. We show that the improvement in survival is seen, possibly to a greater extent, in those with no cardiac involvement and early to moderate cardiac involvement. A rapid response may avoid organ progression in early-stage patients. Due to a lack of data on organ involvement each month, this study has a limitation in being unable to report on organ progression at earlier time points.

The international amyloidosis society consensus guidelines validated response criteria in 2012 (updated in 2020) and showed that patients with a complete response (aCR) had better overall survival than those with VGPR or lesser responses [3, 13]. Over the last few years, we and others have shown that a deeper FLC response is also associated with better outcomes based on reaching dFLC <10 mg/L [4] or iFLC of <10 mg/L [5] and < 20 mg/L [21], respectively. In the current cohort, we found no difference in the outcomes of early CR vs VGPR patients. We also analysed the data in this study based on the suppression of the amyloidogenic light chain (either dFLC <10 mg/L or iFLC <20 mg/L) for patients with or without aCR, showing significant improvement in survival for patients where the FLC was below the dFLC/iFLC threshold. The difference between these data and previous publications needs to be explored further in collaborative studies but may be related to use bortezomib in all patients in the current cohort compared to a smaller proportion in previous publications [21]. These data suggest that depth of FLC response is important in outcomes and additionally utility, if any, of lower dFLC or iFLC in addition to aCR should be formally validated.

In the current data, only 1/3^rd^ of the patients in our study had achieved a deep response at 1 month with standard Bortezomib-based therapy; therefore, there is a need for more effective and rapidly acting treatments to improve the current standard of care. In the Andromeda phase III prospective trial of CyBorD with or without daratumumab [22, 23], the median time to ≥ VGPR was 17 days for the CyBorD with Daratumumab compared to 25 days for the CyBorD combination. Whilst the current data from this study do not show any survival benefit, and longer-term follow data are awaited with interest. Until more effective combinations become routinely available, the impact of early response on modification of therapy needs to be considered cautiously. These (and previous data) are very clear that patients with suboptimal early response have worse outcomes. The crucial prospective data on the impact of early change in therapy is lacking. In the UK, current guidelines and practice recommend switching therapy in non- or suboptimal responders at three months [24, 25]. These data have two observations. Firstly, patients who have a less deep response at 1 month (not yet reached a VGPR) have a significantly poorer outcome, and secondly, the depth of responses improves over time. Switching therapy too early may mean the best benefit is not obtained and, potentially well-tolerated therapies are abandoned for second-line regimes. The increasing cost of therapy means that decisions also have a health-economic angle in most health care systems limiting access to therapies. On the other hand, waiting two or three months in anticipation of a better response leads to loss of vital time while the proteotoxic and amyloidogenic pathway remains active—an argument in favour of early switching. In a complex disease like AL, decision making needs to be much more nuanced and individualised with attention to the patient’s clinical status and the trajectory of the FLCs. Our practice now is to measure serum-FLCs frequently (once a week at least for the initial cycles) and consider therapy modification for those cases where a partial response is not achieved by 1 month and, for those with >PR at one month, where patients have <VGPR by 2 months.

The present data needs to be interpreted within the limitations of the study. This is a retrospective cohort, and we did not assess the full organ function every month, and there is limited baseline bone marrow information. All patients were treated by local hospitals. Although treatments were given with nationally agreed protocols based on multidisciplinary advice from the National Amyloidosis Center, the doses of the individual chemotherapy agents were at the discretion of the treating teams, based on the patient’s clinical status. There is no data on the impact of early switching of therapy on outcomes.

In conclusion, the results from the present study show that patients achieving an early and deep haematologic response have improved survival in AL amyloidosis. Crucially, the deep early responses improve outcomes across all disease stages. The impact of this early response seems to be driven by the reduction of serum-FLCs. Frequent FLC measurement and early assessment for treatment changes are important. Regimens capable of inducing a rapid and profound light chain response are on the horizon and need early adoption. The impact of switching treatment early in slow responders needs further prospective evaluation.

## Supplementary information

Supplementary Appendix
