# Supplementary material for: Impact of early response on outcomes in AL amyloidosis following treatment with frontline Bortezomib
Source: Blood Cancer J. 2021 Jun 21;11(6):118. doi: 10.1038/s41408-021-00510-7 (PMC8217489; doi:10.1038/s41408-021-00510-7)
Supplement: Supplementary file 1 — Supplementary Appendix [file 41408_2021_510_MOESM1_ESM.docx]

**Supplementary Appendix**

**Impact of the speed of response on outcomes in AL amyloidosis following treatment with frontline Bortezomib**

**Table of Contents**

Patients and Methods Page 2

Figure Legends Page 3

Figure SA1 Page 5

Figure SA2 Page 5

Figure SA3 Page 6

Figure SA4 Page 6

Figure SA5 Page 7

Figure SA6 Page 7

**Patients and Methods**

The ALchemy study is an ongoing prospective observational study of newly diagnosed AL amyloidosis seen at the UK National Amyloidosis Centre (NAC). ALchemy study is approved by the relevant institutional review board and all patients provided written informed consent per the declaration of Helsinki. All patients are seen at the NAC at baseline, and then at least every six months for a comprehensive assessment. All investigations were done at the UK National Amyloidosis Centre, where data was collected and analysed. Patients were treated at their local centres as per nationally agreed protocols. Patients were treated with intravenous Bortezomib until 2013, after which time subcutaneous Bortezomib was used. All decisions for any change in treatment were made with input from the multidisciplinary team at the UK National Amyloidosis Centre and considered the patients’ haematologic response (or progression), degree of organ damage, whether there was an improvement in organ function and functional status. However, dose modifications of chemotherapy agents, steroid doses and the number of treatment cycles were at the discretion of the locally treating physician.

Diagnosis of AL was confirmed with biopsy immunohistochemistry, and proteomic analysis was performed where required. All patients underwent serial biochemical tests for organ function, serum-free light chains, serum, and urine protein electrophoresis/immunofixation, cardiac biomarkers, echocardiography, or cardiac MRI (unless contraindicated). As per the standard follow up protocol, serum-free light chain (FLC) and serum monoclonal protein levels (when present) levels were monitored at the beginning of each cycle of chemotherapy.

**Figure Legends**

**Figure SA1:** Kaplan-Meier curve showing overall survival based on the haematologic response at 3 months (ITT Cohort) There was no significant difference in survival between CR and VGPR at 3 months - median not reached in both groups (p=0.230). 94%, 90%, 71% & 56% of patients with a CR at 3 months were alive at the end of 1, 2,

5 & 10 years from diagnosis, respectively. 92%, 87%, 67% & 52% of patients with a VGPR at 3 months were alive at the end of 1, 2, 5 and 9 years from diagnosis, respectively. In contrast, the median OS of patients with PR and NR at 3 months was 50 months (95% CI 30.51-69.48 months, p<0.005), and 4 months (95% CI 2.85-5.14 months, p<0.005), respectively.

**Figure SA2:** Kaplan-Meier curve showing overall survival based on the haematologic response at 6 months (ITT Cohort). There was no significant difference in survival between CR and VGPR at 6 months - median not reached in both groups (p=0.070). 95%, 91%, 74% & 63% of patients with a CR at 6 months were alive at the end of 1, 2,

5 & 10 years, respectively. 95%, 90%, 66% & 52% of patients with a VGPR at 6 months were alive at the end of 1, 2, 5 and 10 years from diagnosis, respectively. In contrast, the median OS of patients with PR and NR at 6 months was 48 months (95% CI 33.91- 62.09 months, p<0.005), and 4 months (95% CI 3.47-4.52 months, p < 0.005), respectively.

**Figure SA3:** Kaplan-Meier curve showing the impact of early versus late responses on overall survival in the 6-month landmark cohort. Patients with an early response (≥ VGPR at 1-month) had a significantly better OS than those with late response (≥ at 6 months)- Median OS not reached vs 74 months (p=0.027). 93%, 87%, 73% & 66% of patients with ≥ VGPR at 1-month were alive at the end of 1, 2, 5 & 10 years from the landmark point, respectively.

**Figure SA4:** Kaplan-Meier curve showing the impact of low dFLC (< 10 mg/l) on patients with CR at 1-month. There was no significant difference in survival between patients with CR and dFLC < 10 mg/l and those with CR and dFLC > 10 mg/l at 1-month- median not reached in both groups (p=0.217).

**Figure SA5:** Kaplan-Meier curve comparing survival in patients with CR versus those with dFLC < 10 mg/l in the 6-month landmark cohort. There was no significant difference in OS between the two groups- median OS not reached in both groups (p=0.617). 93%, 88%, 74% and 63% of patients with CR were alive at the end of 1, 2, 5 and 10 years from the landmark point, respectively. 95%, 91%, 71% and 61% of patients with dFLC < 10 mg/l were alive at the end of 1, 2, 5 and 9 years from the landmark point, respectively.

**Figure SA6:** Kaplan-Meier curve comparing survival in patients with CR versus those with iFLC < 20 mg/l in the 6-month landmark cohort. There was no significant difference in OS between the two groups- median OS not reached in both groups (p=0.233). 93%, 88%, 74% and 63% of patients with CR were alive at the end of 1, 2, 5 and 10 years from the landmark point, respectively. 94%, 91%, 77% and 67% of patients with iFLC < 20 mg/l were alive at the end of 1, 2, 5 and 9 years from the landmark point, respectively.
